# Why Are Patients Unhappy with Their Healthcare? A Romanian Physicians’ Perspective

**DOI:** 10.3390/ijerph19159460

**Published:** 2022-08-02

**Authors:** Bianca Hanganu, Irina Smaranda Manoilescu, Cristian Paparau, Laura Gheuca-Solovastru, Camelia Liana Buhas, Andreea Silvana Szalontay, Beatrice Gabriela Ioan

**Affiliations:** 1 Department of Forensic Medicine, Faculty of Medicine, Grigore T. Popa University of Medicine and Pharmacy, 700115 Iasi, Romania; irina.manoilescu@umfiasi.ro (I.S.M.); beatrice.ioan@umfiasi.ro (B.G.I.); 2 Dambovita County Forensic Medicine Service, Targoviste Emergency County Hospital, 130086 Targoviste, Romania; cristianpaparau@yahoo.com; 3 Department of Clinical Dermato Venereology, Faculty of Medicine, Grigore T. Popa University of Medicine and Pharmacy, 700115 Iasi, Romania; solovastru.gheuca@umfiasi.ro; 4 Department of Morphological Disciplines, Faculty of Medicine and Pharmacy, University of Oradea, 410087 Oradea, Romania; cameliabuhas@yahoo.com; 5 Department of Psychiatry, Faculty of Medicine, Grigore T. Popa University of Medicine and Pharmacy, 700115 Iasi, Romania; andreea.szalontay@umfiasi.ro

**Keywords:** medical malpractice complaints, physicians’ perspective, reasons, relational aspects

## Abstract

Background: Medical professional liability complaints are not triggered by a single factor, but rather by multiple factors, each having more or less implications, such as the characteristics of the physician, the medical system, the patients, the complexity of their pathology, and the inherent limits of medicine. Knowledge about the factors that initiate the complaint procedure is essential to identify the targeted measures to limit their prevalence and impact. The purpose of this study was to identify the reasons behind the malpractice complaints and the factors that may influence the initiation of complaints by the patients. Material and Methods: This study was conducted using an online questionnaire, addressed to Romanian doctors, with questions about the reasons for patient dissatisfaction and complaints, the factors that predispose a physician to being complained against, and the protective factors against patient complaints. Results: The study group included 1684 physicians, of whom 16.1% were themselves involved in a complaint, and 52.5% knew of a colleague who was complained against. The opinions of the participants regarding the reasons for the complaints, the predisposing factors to complaints, and the factors that contributed to the reported incident showed a strong link between professional liability complaints and the physician–patient/patient’s family relationship. The relationship between fellow physicians is additional to this. Conclusion: This study reveals that the improvement in the relational aspects of medical practice (physician–patient relationship and relationship between physicians) has the highest potential to decrease the number of malpractice complaints. Its practical relevance is related to the need for training physicians in the relational aspects of medical practice during academic years and throughout their career.

## 1. Introduction

When things go bad in medical practice, the responsible party is not always the physician alone, because failure is rather the effect of multiple factors, which create a web that includes various components related to the physician, the medical institution, the medical system [1,2], the patient, and the inherent limits of medicine. These elements were included in the Swiss cheese model issued by James Reason [3], initially to explain and prevent industrial accidents, and later suggested to be applied in medicine. Being aware of this model and, consequently, detecting what can go wrong at each level allows the development of possible methods to overcome the wrong and, subsequently, the avoidance, or at least the decrease in the risk, of failure in medical practice [4].

The medical profession is not risk-free, and although risks are recognized by the medical community, they must be clearly explained to the patients before they sign and thus accept them through the informed consent form. The problem occurs when there is discordance between what the patients consider to be a medical mistake and what is indeed a medical mistake, as there are many cases when the medical act fails despite the fact that the physician followed the standards of good practice [5].

Patient complaints about physicians’ professional liability have registered an upward trend over time in countries around the world. In the United Kingdom, the number of patient complaints against general practitioners increased over twofold in a five-year period, from 2007 to 2012 [6]. On the Asian continent, in China, Wang et al. [7] reported that the number of claims in civil courts increased significantly, from 75 in 2010 to 6947 in 2014. In Saudi Arabia, over a period of 10 years—between 1999 and 2008—the number of complaints increased from 440 to 1356 [8]. In Romania, research on this subject is scarce. The study published by Dumitrescu [9] in 2019 showed that the number of medical malpractice claims filed annually in court increased from 8 in 2008 to 65 in 2017, with a total of 331 between 27 November 2007 and 1 April 2018. Out of court, a total of 153 complaints were analyzed between 2006 and 2019 in the eight counties of the Moldova region in Romania [10].

Investigating the reasons and factors that fuel the medical malpractice complaints and the consequent identification of targeted measures to limit their prevalence is an indirect way to prevent the impact of complaints against physicians.

The aim of this study was to identify the potential factors related to the initiation of complaints regarding the professional liability of physicians in Romania.

## 2. Materials and Methods

The authors conducted a prospective quantitative study based on a questionnaire applied online through the GoogleDocs platform. The questionnaire was available for completion for three weeks, between 4 May and 24 May 2020. The questionnaire was constructed after performing a thorough analysis of the scientific literature for the identification of similar studies [11,12,13], to which we added the results obtained during a quantitative study performed prior to the current one by members of the research team [10]. The questionnaire included 88 questions (both open and closed), structured into three sections:

- A section with general questions, addressed to all physicians practicing in Romania (socio-demographic, educational, professional, and general aspects related to the risk of malpractice complaints);
- A section with specific questions for physicians who knew of a fellow physician who was complained against (reasons for the complaint and the impact of the complaint on them and their colleagues);
- A section with specific questions for the physicians who themselves were complained against (the reasons for the complaint, the factors that contributed to the complaint, and the impact of the complaint on the complained physician).

Given that the questionnaire was applied online through the GoogleDocs platform, it allowed for a large number of doctors across the country and from various specialties to access it.

Prior to distributing the link in the online environment, the questionnaire was pretested in several phases on a limited number of doctors (n = 10 at the final pretesting phase), being improved based on their feedback and estimating a completion time of about 15 min for those who do not know anyone who received a complaint and 30 min for the other two categories of participants.

The sample of participants was random and heterogeneous, extracted from the population of physicians who are part of the Romanian College of Physicians. In 2018, it consisted of 60,585 members. Inclusion criteria: questionnaire being fully filled in, participants being members of the Romanian College of Physicians. Exclusion criteria: empty or incomplete questionnaire, other professionals than physicians. The sample size was calculated according to the formula:

n = N × X/(X + N − 1), where N is the population size and X = Z^2^ × p × (1 − p)/E^2^,

where Z (=2.32) is the coefficient corresponding to the confidence level of 98%, p is the population proportion (in our case 50%), and E is the margin of error (in our case is 2.8%).

Before proceeding to answering the questions, the potential participants had the option to read detailed information about the study: aim and objectives, scientific use of the results, confidential and anonymous character of the data the participants provided, and the voluntary character of the participation— information that was included on the first page of the questionnaire.

This article addresses the following aspects related to the risk of malpractice complaints, as perceived by the physicians: the reasons for the patient’s dissatisfaction and for the complaint against the physician (from the experience of the complained fellow physicians and of the physicians who were themselves complained against), the protective factors as well as the factors that predispose a physician to be complained against, and the factors that contributed to the incidents for which the physicians were complained against (from their own perspective).

The collected data were analyzed using SPSS software, v 20 (IBM corp., Armonk, NY, USA), using frequency analysis and ANOVA with repeated measures.

This study is part of a doctoral research that received the approval of the Research Ethics Commission of the Grigore T. Popa University of Medicine and Pharmacy of Iasi, no. 16434/30 July 2019.

## 3. Results

### 3.1. The Structure of the Group of Participants

The group of participants included 1684 physicians. Of these, 16.1% (N = 271) were the subject of medical malpractice complaints and 52.5% (N = 884) knew of a colleague who was involved in such a complaint. Of these, 71.0% (N = 1196) were women, 52.1% (N = 878) were senior physicians, and 90.4% (N = 1522) worked in urban areas. The mean age of the participants was 44.77 years (±10.98 years), and the mean work seniority in the profession was 18.09 years (±11.53 years). The complete socio-demographic and professional characteristics of the participants are presented in Table 1.

### 3.2. The Most Common Reasons for Patient Dissatisfaction

**Figure 1:**
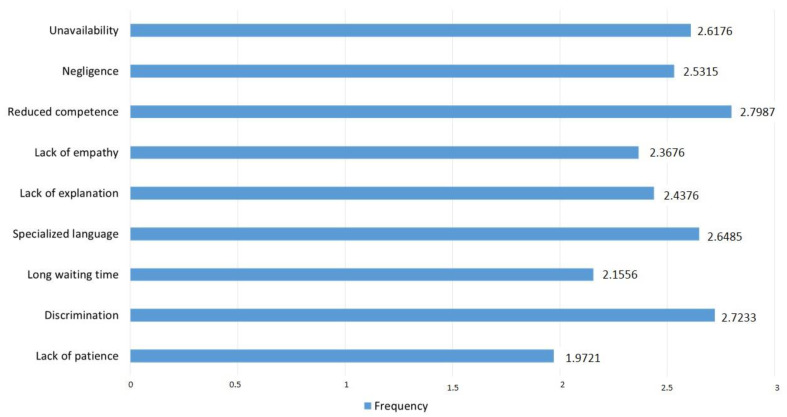
Reasons for patient dissatisfaction.

The ANOVA analysis with repeated measures showed significant differences when tested for the reasons that make the patient unhappy with the medical system (*p* < 0.01). For this question, we used the Likert scale with 5 points. Figure 1 presents all the reasons that were tested and the frequency scores according to the options of the participants. Low scores indicate a high frequency of motives, while high scores indicate a low frequency. The three most common reasons selected by the physicians participating in this study were the following: the physician’s lack of patience to listen to the patient, long waiting times to receive the consultation, and the physician’s lack of empathy.

### 3.3. Factors That Predispose a Physician to Being Complained against by a Patient

**Figure 2:**
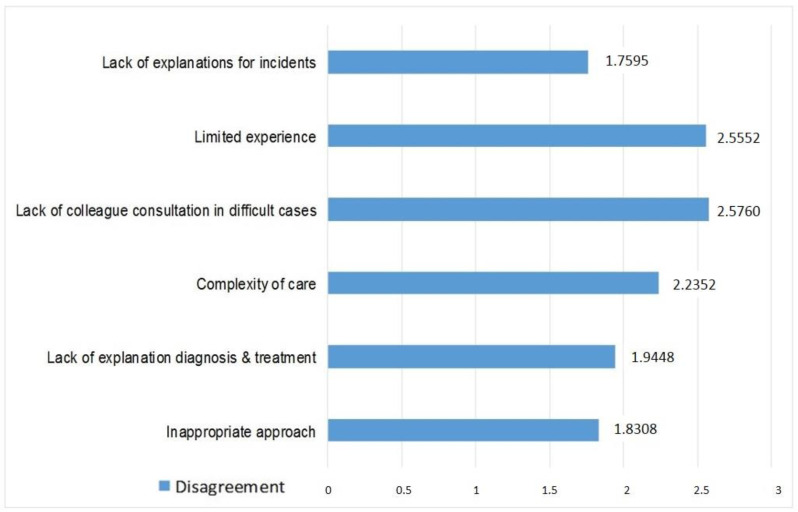
Factors that predispose physicians to complaints.

The ANOVA analysis with repeated measures showed significant differences when tested for the importance of six factors that predispose a physician to complaints (*p* < 0.01). For this question, we used the Likert scale with 5 points. Figure 2 presents all the factors that were tested and the frequency scores according to the options of the participants. Low scores indicate a high frequency factor, while high scores indicate a low frequency factor. In the opinion of the participants, the three most important factors were the following: lack of explanation in case of occurrence of an adverse event/incident, unsuitable approach from the doctors, or an unsuitable manner in which they communicate with their patients, to which offering no explanations regarding the diagnosis and treatment was added. These three factors were assessed by the participants as being significantly more important than the other three (complexity of care, lack of consultation with other colleagues on difficult cases, and limited experience in the field), which were evaluated around the neutral point in terms of importance. Additionally, the limited experience in the field and the lack of consultation with other colleagues were evaluated as representing the factors closest to the neutral point as important for the formulation of a complaint by the patient.

### 3.4. Reasons for the Complaints against the Participants’ Colleagues

The frequency analysis for the reasons stated by physicians who had a colleague involved in a complaint indicated the following most common reasons that triggered the complaint: complications related to treatment (33%), issues related to doctor–patient relationship (32.4%), wrong treatment (23.4%), and wrong diagnosis (20.5%). The least frequently cited reasons were: breach of confidentiality (1.2% of cases), deficiencies in completing the observation sheet (4.5%), and deficiencies in obtaining informed consent (4.6%). The complete results are shown in Table 2.

### 3.5. Reasons for the Complaints against the Participants Themselves

In the group of physicians who were complained against by their patients, the hierarchy of reasons were slightly different compared to the reasons reported by the participants who knew of a colleague who was involved in a complaint. In this group, the most common reasons were: complications related to treatment (24.7%), complaint being suggested to the patients by other physicians (20.7%), and dysfunctions of the physician–patient relationship (20.3%). The least common reasons were: breach of confidentiality (0.7%), deficiencies in obtaining informed consent (1.1%), and issues related to the completion of the observation sheet (1.8%). The complete results are shown in Table 2.

### 3.6. Elements That Had a Role in the Occurrence of the Incident/Adverse Event That Triggered the Patients to Complain

The physicians that were themselves complained against stated that the elements which had a role in the occurrence of the incident or the adverse event that triggered the complaint were the following: difficult relationship with the patient (59.0%), patient comorbidities (48.3%), lack of protocols or guidelines (27.3%), and fatigue (22.5%). Less frequently invoked were: stress caused by problems in the family (1.8%), non-compliance with the protocol or guidelines (3.3%), and stress caused by problems with the superiors (8.5%).

### 3.7. Elements That Could Protect Physicians against Malpractice Complains

**Figure 3:**
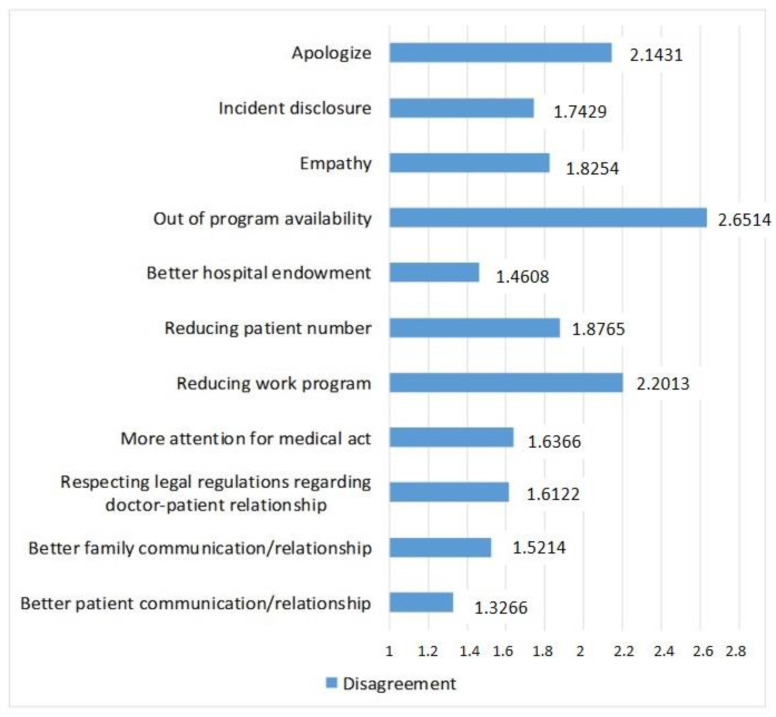
Elements that could protect against a malpractice complaint.

The ANOVA analysis with repeated measures showed significant differences when tested for the 11 types of elements that could protect physicians from being complained against (*p* < 0.5). For this question, we used the Likert scale with 5 points. Figure 3 presents all the elements that were tested and the frequency scores according to the options of the participants. Low scores indicate a high frequency of the factors, while high scores indicate a low frequency. The elements that proved to be the most important were the following: improvements in the communication/relationship between the physicians and the patient, better logistics for the medical facilities, and improvement in the communication that takes place between the physician and patient’s family. These three factors were assessed by the participants as being significantly more important compared to the other eight.

## 4. Discussion

The results of our study indicate a strong link between the professional liability claims and the relationship between the physician and the patient or his/her family. Thus, the opinions of the study participants regarding the patients’ dissatisfaction and the predisposing factors to the complaints, the reasons for the complaints, and the factors that contributed to the reported incident include aspects regarding this relationship. Consequently, among the factors that could contribute to decreasing the possibility of complaints, the physicians participating in the current study placed the improvement of the relationship with the patient first. To these aspects, the relationship between fellow physicians, and the physicians involved in a complaint who frequently highlighted the suggestions coming from other physicians as the reason for the complaint were added.

If we consider the big picture, these results are consistent with other studies published in the literature. Nevertheless, the underlying factors triggering these deficiencies in doctor–patient relationship and the manner in which they contribute can vary across cultures. Physicians who do not take into account the feelings and the concerns of their patients, who limit the information they offer to their patients, and the physicians who rush their patients are more predisposed to complaints in comparison to their fellow physicians who are more attentive to these aspects [15]. In addition, Chiu [15] and Posner et al. [16] highlighted that certain attitudes, such as lack of empathy and lack of attention to the concerns of the patients and their families, poor information, and poor listening, are factors that predisposed the physician to complaints from their patients. Klepacka and Bakalarski [17], when analyzing the confidence in doctors among general population, found that one of the biggest disadvantages was bad contact from the doctors with the patients. When patients are allowed to speak and express their concerns, they can reveal information that is essential for the diagnosis process and the evolution of the treatment. Their speeding up by the physician can intimidate them, limiting the information they will provide, which can consequently lead to errors in diagnosis and treatment [18]. Charles et al. [19] state that physicians with no history or insignificant history of complaints reported spending more time with patients compared to those who were complained against more than once. The coherent combination of professionalism with attitudes that express empathy, care, and kindness can counteract the patient’s decision to file a complaint in the event of a medical failure [20].

Complications of the medical act ranked first in terms of the reasons for the complaint in both categories of participants: those who knew of a colleague who was involved in a complaint and those who were themselves the subject of a complaint. This result coincides with that obtained by us in a previous study conducted in the region of Moldova, Romania, which looked into the complaints submitted by patients for out-of-court settlement [10].

The complexity of the medical act imposed by the patient’s complex pathology often puts physicians in front of difficult situations, meant to test their professional knowledge and skills at the highest level, and the results of the therapeutic interventions are not always commensurate with the effort. The failure or success of the medical interventions is dependent on a multitude of human and non-human factors, so it is unlikely that these complex situations, often followed by complications, will disappear completely from medical practice. However, every effort must be made to minimize these failures by providing a high level of patient safety [21].

The stage of informing the patient has an important role in preventing complaints when complications that are recognized by the international medical community occur. At this stage, the physician informs the patient before the latter signs the informed consent form, so that the patient accepts the medical intervention fully aware of the risks to which he/she will be exposed, apart from the benefits of a successful specific intervention [11]. Veerman et al. [22] examined 245 complaints involving informed consent issues and found that 67% of the cases were related to risks or complications of treatment that patients had not been informed of prior to the procedure.

In order to prevent complaints when unforeseen complications occur, with or without the fault of the physician, the essential role is still related to communication with the patient [23], an idea also supported by the physicians participating in our study. Thus, the lack of explanations when an incident occurs is considered an important factor that predisposes the physician to complaints, and better communication and relationship with the patients and their families are factors that can reduce the risk of malpractice complaints. The study performed by Popa et al. [24], who evaluated the Romanian patients’ perception of healthcare professionalism, shows that one of the five unpleasant aspects while being admitted to the hospital was the inefficient communication between the doctor and the patient in terms of the lack of information during the informed consent process and showing respect to the patients.

As mentioned earlier, failure in the medical activity involves more than the physician. The physicians participating in our study agreed that better medical facilities could reduce the risk of complaints that may be related to diagnostic and treatment errors. In order to be able to perform their duties properly and for the interpenetration of professional knowledge and skills to achieve the highest possible success rate, the physicians must benefit from appropriate equipment and favorable working conditions [21].

Communication is closely linked both to the initiation of complaints [25] and to their prevention, even when medical errors occur [26]. On the one hand, the participants in our study agreed that a better relationship with the patient and his/her family could reduce the risk of complaints, and on the other hand, they felt that omitting to explain the reasons for which an incident occurred, deficiencies in approaching or communicating with the patient, as well as the lack of explanations about the diagnosis and treatment are important elements that make physicians prone to receiving complaints. Effective communication allows the physician to inform the patients more clearly about the risks, giving them the opportunity to judge and decide for themselves whether or not to take them [27]. Griffen et al. [28] showed that 34% of malpractice complaints against general surgeons were caused by poor communication with the patients and their family members. Hickson [29] also found that the lack of relational skills was a predictive risk factor for complaints, and Fishbain [30] referred to the tone of the physician’s voice when addressing the patients, the harsh tone being related to the history of complaints.

It is essential, correct, and moral for physicians to communicate when an incident of a medical act occurs, the patients having the right to receive explanations for the damage they suffered, regardless of the circumstances in which it occurred [31,32]. There are studies showing that early [33] and complete [33,34] disclosure of incidents increases patients’ confidence in their physicians, helps them make decisions about the next steps in treatment, overcomes emotional trauma more easily [31], and extinguishes the desire to complain [33]. It is well recognized that patients’ confidence in their physicians is an important step for the success of a medical act [17]. However, many physicians are still reluctant to disclose adverse events to patients [35], especially when they are not very obvious [31,33,36], fearing that the disclosure will adversely affect the physician–patient relationship and will be followed by malpractice complaints [37]. Studies targeting patients who experienced adverse events showed that a major trigger for complaints was the physicians’ lack of honesty and their arrogant attitude, the tendency to avoid and postpone discussions after the incident [15], superficial explanations [38], the lateness with which the explanations were given, or even not explaining at all [39].

In our study, the second most frequent reason related to patient complaints, according to the responses of the participants who were directly involved in the complaints, was the suggestion made by another physician. This result is similar to a previous study by the authors of this article [40] and sounds an alarm about two important issues: first, ensuring that the criticized physician has actually made a mistake—without firing hasty conclusions, and second, the manner in which the mistake of another physician should be revealed to the patient [41]. Most of the time, physicians tend to denigrate their colleagues in front of the patients from the first moment, without carefully analyzing the situation. For example, McDaniel et al. [42] found that physicians often made critical comments about their colleagues even when they were not wrong. It is important for a physician to make sure that the colleague was wrong, and then to identify the best way of informing the patient. Physicians also need to be trained in this field, given that, nowadays, medical practice requires numerous specialists from various institutions who take care of the same patient [41].

Treatment error and diagnostic error occupy the third and fourth places, respectively, in frequency among the reasons for which the colleagues of the physicians participating in our study were complained against, these two reasons having very close values (23.45% and 20.50%, respectively). Diagnostic errors often lead to treatment errors, and both are very costly for the healthcare systems and can result in significant risks to the patient’s health [43,44]. In their study, Gualniera et al. [44] identified that treatment error occupied the first place on the list of reasons leading to complaints and was found in 71.2% (out of 240) of the cases from the out-of-court analysis, and 62.6% (out of 187) of the cases from the court. In comparison, the diagnostic error was identified in 10.8% of the cases analyzed out-of-court and in 23% of the cases analyzed in the court [44]. Knaak and Parzeller [45] analyzed 232 court decisions and found that the most common reasons for complaints (in 67.2% of cases) were the incorrect administration of treatment (invasive administration, wrong dosage, and inappropriate time for administration) and wrong indication of treatment (in 37.1% of cases), followed by a wrong diagnosis (in 31.5% of cases). In their 15-year retrospective study (2000–2014), Wu et al. [46] found that 50% of the 84 malpractice claims reviewed by the Taiwan Supreme Court were for improper treatment (indication, dosage, and timing errors). Gupta et al. [47] found that the number of complaints for wrong diagnosis decreased in the USA between 1999 and 2011, a possible explanation being the progress made in the field of imaging. These findings can be put in line with the results of our study, which indicates that physicians consider that better endowment of hospitals could reduce the risk of complaints.

The physicians participating in our study reported that, in addition to the relationship with the patients and comorbidities of the patients, fatigue and lack of clinical protocols were other factors that led to the incident for which they were complained against. Fatigue can be the consequence of overloading physicians and the Romanian medical system, with a discrepancy between the severity of cases and the type of hospital accessed by the patients. It is especially the case in the overloading of emergency services for non-emergency cases, and in the county/regional and university hospitals with cases that can be resolved in smaller hospitals or by a family physician [10]. The high workload in inadequate conditions—both in terms of equipment and support from colleagues or superiors—puts undue pressure on the physicians, thus even well-trained physicians risk making mistakes [21,48]. Furthermore, the system of remuneration based on the number of patients (applicable to private medical institutions and systems) exacerbates the overload, negatively impacting the physician–patient relationship and the quality of care [49]. A study performed in New Zealand, which included 301 anesthesiologists and analyzed the fatigue errors related to the work program, concluded that more than 80% of participants declared a connection between fatigue and errors they made [50]. The study performed by Landrigan et al. [51] revealed that working more than 24 continuous hours increased the number of errors made by resident physicians by 35.9%, compared to the number of errors they made during the shifts of a maximum of 16 h.

When there are no medical protocols, physicians are exposed to a higher risk of making mistakes and of being without defense in the event of complaints. As a result, some physicians could try to find the alternative of defensive practices, with negative consequences for patients, physicians, and the medical system in general [52,53].

All complex systems, such as the medical system, benefit from many protective barriers between the patient and the failure of the medical act, a situation that can be described by using the Swiss cheese model. Some protective barriers can be natural (e.g., physical barriers), others can be artificially designed (e.g., different sensors that trigger alarms), others are represented by the presence of specialists (e.g., surgeons), and others are administrative (e.g., protocols and guides) [3]. In an ideal world, the slices of cheese would be compact. In reality, each slice has defects, similar to the holes in Swiss cheese, which close, open, and change their location constantly [3,54]. Defects may occur due to a combination of active failures (incorrect execution of a well-established protocol, omission of a protocol step, conscious—but not malicious—non-compliance with the protocol) and latent conditions—which modulate closure, opening, and location of the holes in cheese slices, and which may be organizational, contextual, diffuse, or design-related (inefficient equipment, malfunctions, poor design of equipment and materials used, inadequate training, inadequate supervision, or inadequate communication) [3,54,55].

In most cases, the existence of these holes is not dangerous: a defect in a barrier can be counteracted by the other slices. However, when the holes in all of the slices line up, a free path is created to cause harm, and the inevitable becomes a reality [3,55].

## 5. Strengths and Limitations

A significant strength of the current study is the fact that we applied the questionnaire at the national level, and we included doctors from various specialties, with various seniorities (i.e., residents, specialists, and senior doctors), both involved and not involved in complaints from patients, which allowed for obtaining a general national frame of the topic. Given the sample size, the results of this study can be generalized to the entire community of physicians in Romania.

A limitation of our study is the fact that the results cannot be extrapolated to other countries, given that cultural factors can play a role in triggering complaints, especially when we consider the doctor–patient relationship.

## 6. Conclusions

An effective relationship between the physician and the patient, in all its aspects (e.g., communication, attitude, empathy), is essential to the success of the medical act and implicitly to the prevention of medical professional liability complaints. This is all the more evident as medical practice is becoming increasingly complex, with particular risks associated with technological progress, which patients need to be informed about, according to their level of understanding. At the same time, the lack of access to advanced technology can lead to diagnostic and/or treatment errors or delays.

Diagnostic errors and treatment errors are closely related to the relationship between the partners in the medical act, to the lack of complete information provided by the patient, and if the physician does not have the patience to listen to the patient or does not allow him/her to express all his/her concerns.

Suggestions for complaints made by other physicians—directly or indirectly through subtleties—sound an alarm about the conditions and the manners in which physicians who observe colleagues’ mistakes should disclose them to the patients.

The results of our study are of particular practical importance, emphasizing the importance of effective communication and the relationship between physician and patient, as well as an effective relationship between physicians caring for the patient. These aspects must be part of the curriculum of medical universities, but also of the continuing medical education of medical professionals.

## Figures and Tables

**Table 1:** Socio-demographic and professional characteristics of the participants *^,1^.

Characteristics	N (%)
**Gender**	
Male	488 (29.0)
Female	1196 (71.0)
**Marital status**	
Single	302 (17.9)
In a relationship	1382 (82.1)
**Children**	
No	543 (32.2)
Yes	1141 (67.8)
**Graduating institution**	
Private	50 (3.0)
Public	1634 (97.0)
**Medical specialty**	
Family medicine	321 (19.1)
Medical	692 (41.1)
Surgical	370 (22.0)
Pediatrics	123 (7.3)
Laboratory	178 (10.6)
**Professional degree**	
Resident	161 (9.6)
Specialist	645 (38.3)
Senior	878 (52.1)
**Teaching activity**	
No	1441 (85.6)
Yes	243 (14.4)
**Area of activity**	
Urban	1522 (90.4)
Rural	88 (5.2)
Both	74 (4.4)
**Type of medical institution I**	
Public	749 (44.5)
Private	482 (28.6)
Both	453 (26.9)
**Type of medical institution II**	
Regional/county hospital	709 (42.1)
Municipality hospital	274 (16.2)
City hospital	101 (5.9)
Polyclinic/ambulatory	525 (31.1)
Private medical office	513 (30.4)
Private hospital/clinic	658 (39.0)
**Type of patients**	
Mostly women	260 (15.4)
Mostly men	62 (3.7)
Women and men equally	1362 (80.9)
**Characteristics**	**M ± SD**
Age	44.77 ± 10.98
Work seniority	18.09 ± 11.53

* This study is part of a larger doctoral research, and the same group of participants was analyzed in a different paper, from a different perspective, published somewhere else [14]. ^1^ Number of answers (N), percentage (%), mean value (M), and standard deviation (SD).

**Table 2:** Reasons for complaints ^1^.

Reason	Colleagues N (%)	Participants N (%)
Treatment complications	292 (33.0%)	67 (24.7%)
Physician–patient relationship	286 (32.4%)	55 (20.3%)
Treatment error	207 (23.4%)	47 (17.3%)
Diagnostic error	181 (20.5%)	53 (19.6%)
Suggested by colleagues	106 (12.0%)	56 (20.7%)
Difficult communication within the medical team	76 (8.6%)	14 (5.2%)
Related to the completion of the medical file	40 (4.5%)	5 (1.8%)
Deficiency in obtaining informed consent	41 (4.6%)	3 (1.1%)
Breach of confidentiality	11 (1.2%)	2 (0.7%)

^1^ Number of answers (N) and percentage (%).

## Data Availability

The data are available, on request, from the corresponding author.
